# A Truncated TIR-NBS Protein TN10 Pairs with Two Clustered TIR-NBS-LRR Immune Receptors and Contributes to Plant Immunity in *Arabidopsis*

**DOI:** 10.3390/ijms22084004

**Published:** 2021-04-13

**Authors:** Yongming Chen, Guitao Zhong, Huiren Cai, Renjie Chen, Na Liu, Wei Wang, Dingzhong Tang

**Affiliations:** 1 State Key Laboratory of Ecological Control of Fujian-Taiwan Crop Pests, Key Laboratory of Ministry of Education for Genetics, Breeding and Multiple Utilization of Crops, Plant Immunity Center, Fujian Agriculture and Forestry University, Fuzhou 350002, China; Chesy6666@163.com (Y.C.); guitaozhong@163.com (G.Z.); caihuiren@163.com (H.C.); ch_rjars@yeah.net (R.C.); liuna20002004@163.com (N.L.); 2 College of Life Science, Fujian Agriculture and Forestry University, Fuzhou 350002, China

**Keywords:** plant immunity, NLRs, TIR, TN, TNL, cell death, ETI

## Abstract

The encoding genes of plant intracellular nucleotide-binding site (NBS) and leucine-rich repeat (LRR) domain receptors (NLRs) often exist in the form of a gene cluster. Several recent studies demonstrated that the truncated Toll/interleukin-1 receptor-NBS (TIR-NBS) proteins play important roles in immunity. In this study, we identified a large *TN* gene cluster on *Arabidopsis* ecotype Col-0 chromosome 1, which included nine *TN* genes, *TN4* to *TN12*. Interestingly, this cluster also contained two typical *TIR-NBS-LRR* genes: *At1g72840* and *At1g72860* (hereinafter referred to as *TNL40* and *TNL60,* respectively), which formed head-to-head genomic arrangement with *TN4* to *TN12*. However, the functions of these *TN* and *TNL* genes in this cluster are still unknown. Here, we showed that the TIR domains of both TNL40 and TNL60 associated with TN10 specifically. Furthermore, both TNL40TIR and TNL60TIR induced cell death in *Nicotiana tabacum* leaves. Subcellular localization showed that TNL40 mainly localized in the cytoplasm, whereas TNL60 and TN10 localized in both the cytoplasm and nucleus. Additionally, the expression of *TNL40*, *TNL60*, and *TN10* were co-regulated after inoculated with bacterial pathogens. Taken together, our study indicates that the truncated TIR-NBS protein TN10 associates with two clustered TNL immune receptors, and may work together in plant disease resistance

## 1. Introduction

The growth environment of plants also contains a range of microorganisms, including viruses, bacteria, fungi, and oomycetes, some of which infect plants to exploit nutrition [1]. In addition to non-host resistance, including natural physical barriers and constitutive synthesis of various enzymes and some secondary metabolites, plants also rely on the innate immune system to defend against pathogens [2,3,4,5]. The innate immune system of plants is mainly divided into two layers. Pattern recognition receptors (PRRs) at the plasma membrane recognize the conserved components of pathogens, pathogen-associated molecular patterns (PAMPs), or microbe-associated molecular patterns (MAMPs), and activate the first layer of immune responses, termed pattern-triggered immunity (PTI) [2,6,7]. The plant PRRs can be classified into two types: receptor-like kinases (RLKs) and receptor-like proteins (RLPs) based on the presence or absence of a kinase domain, respectively [7,8]. The well-studied RLKs in *Arabidopsis* include FLAGELLIN SENSING 2 (FLS2) and EF-TU RECEPTOR (EFR), which recognize conserved 22-amino acids peptide of bacterial flagellin (flg22) and the bacterial elongation factor TU (EF-TU), respectively [9,10,11,12,13,14].

Some host-adapted pathogens can secrete effectors that contribute to pathogen virulence into plant cells to interfere with plant immunity. Plants have evolved corresponding intracellular nucleotide-binding site (NBS) and leucine-rich repeat (LRR) domain receptors (NLRs), which are also called resistance (R) proteins that specifically recognize some effectors directly or indirectly and activate the second layer of immune responses, termed effector-triggered immunity (ETI) [2,15,16]. Compared with PTI, the response of ETI is often more intense and rapid, usually followed by hypersensitive responses (HR), a type of programmed cell death at the infection site [17,18].

In plants, the typical NLR protein often contains a specific N-terminal domain, a conserved NBS domain, and a highly variable LRR domain at the C-terminus [19,20]. The specific N-terminal domain is typically a coiled-coil (CC) domain, a Toll/interleukin receptor (TIR) domain or a RESISTANCE TO POWDERY MILDEW 8 (RPW8)-like domain. According to the difference of their N-terminal domain, NLRs are mainly divided into three subfamilies: CC-NBS-LRR (CNL), TIR-NBS-LRR (TNL), and RPW8-NBS-LRR (RNL) [15,18,21]. In addition to the typical NLRs containing all three characteristic domains, there are also some truncated NLRs [22,23]. For example, 21 truncated TIR-NBS (TN) proteins lacking C-terminal LRR domains are identified in *Arabidopsis* ecotype Col-0 [18,21,22,23]. Several recent studies demonstrated that TNs play an important role in plant immunity as well. For instance, the loss of function of *TN8* and *TN11* genes leads to enhanced susceptibility to *Pseudomonas syringae* pv. *tomato* (*Pto*) strain DC3000 [24]. Ectopic expression of the TIR domain of TN2 triggers cell death in tobacco. The autoimmune responses in *exo70B1* mutants are caused by the activation of TN2 and co-expression EXO70B1 with TN2 can suppress the TN2-induced cell death in tobacco, indicating that TN2 may monitor EXO70B1 integrity [25,26]. Moreover, TN2 interacts with CALCIUM-DEPENDENT PROTEIN KINASE 5 (CPK5) and stabilizes the kinase activity of CPK5 [27]. TN13 is reported to be required for resistance against *Pto* DC3000 lacking the effectors AvrPto or AvrptoB [28]. The gain-of-function mutant of *TN1*, also known as *CHILLING SENSITIVE 1* (*CHS1*), displays chilling sensitivity at low temperatures, and the chilling sensitive phenotypes of *chs1-2* mutants at low temperatures are dependent on the full-length TNL protein SUPPRESSOR OF *chs1-2*, 3 (SOC3) [29,30,31]. SOC3 is found to associate with TN1 and TN2, respectively, to monitor the homeostasis of *Arabidopsis* E3 ubiquitin ligase SENESCENCE-ASSOCIATED E3 UBIQUITIN LIGASE1 (SAUL1). The over-accumulation of SAUL1 is monitored by the SOC3-TN2 pair, while SAUL1 disappearance is guarded by the SOC3-CHS1 pair [32,33].

Interestingly, *SOC3* and *CHS1*/*TN2* show a head-to-head orientation arrangement on the gene cluster [31,33]. The head-to-head clusters are frequently found for the plant *NLR* genes. For instance, *RPS4*-*RRS1* from *Arabidopsis* is one of the best characterized head-to-head *NLR* pairs. RPS4 interacts with RRS1 through their TIR domains [34]. The WRKY domain of RRS1 was shown to be able to recognize different effectors to activate ETI responses. The ability of RPS4-RRS1 to activate the defense responses depends on both proteins being present and functional [35]. *CHS3* and *CSA1* is another head-to-head *NLR* pair in *Arabidopsis* [36]. Autoimmunity in the *chs3-2D* mutants requires the presence of a functional CSA1 protein [37], suggesting a cooperative activation mechanism requiring both CSA1 and CHS3. In rice, the head-to-head *NLR* pairs are also identified, such as *RGA4*-*RGA5* pair [38] and *Pik-1*-*Pik-2* pair [39], indicating that the head-to-head arrangement of *NLR*s appears to be a conserved mechanism across species. The possible reason for this arrangement is that the paired *NLRs* are beneficial in co-regulation and co-evolution. Therefore, the investigation of the interaction between NLRs in gene clusters may reveal the functional link of unobserved NLR protein pairs [40].

In this study, through an analysis of the *Arabidopsis* ecotype Col-0 genome sequences, we found a large *TN*-type gene cluster on chromosome 1. This cluster contains nine *TN* genes named *TN4, TN5, TN6, TN7, TN8, TN9, TN10, TN11,* and *TN12*. Interestingly, two full-length *TNL* genes, *TNL40* and *TNL60*, were located at this gene cluster. Here, we show that TN10 interacted with TNL40 and TNL60. Transient expression of both TIR domains of TNL40 and TNL60 triggered cell death in *N. tabacum*. TNL40 localized in the cytoplasm, while TN10 and TNL60 localized in both the cytoplasm and nucleus. The co-expression of *TNL40*, *TNL60*, and *TN10* was observed after the treatment of *Pto* DC3000 and *Pto* DC3000 *avrRpt2*. Our study suggested that TN10 may contribute to plant immunity by associating with both TNL40 and TNL60.

## 2. Results

### 2.1. The TN4-TN12 Gene Cluster Contains Two Typical TNL Genes: TNL40 and TNL60

**Figure 1:**
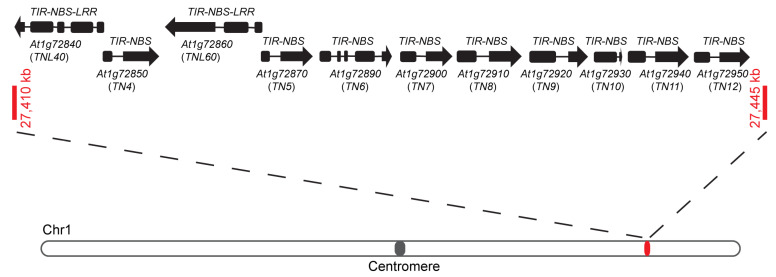
Schematic description of the largest *Toll/interleukin receptor* (*TIR*)*-nucleotide binding site* (*NBS*)*-type* (*TN*) gene cluster with two typical *TIR*-*NBS*-*leucine-rich-repeat* (*TNL*) genes, *TNL40* and *TNL60*.

The genome of *Arabidopsis* ecotype Col-0 contains 21 *TN* genes that are encoded by 20 genes and 1 pseudogene [23]. We found that the largest *TN* cluster was located on chromosome 1. As shown in Figure 1, this gene cluster contained nine *TN* genes, *TN4-TN12*, and all of these *TN* genes were arranged in the same direction. It was reported that *TN10* and *TN11* shared the highest homology with *TN8* and *TN9,* respectively [23]. Among these *TN* genes, the transcripts of *TN7* and *TN10* were reported to be higher than those of other *TNs* [22]. Interestingly, we observed that there were two full-length *TNL* genes, *TNL40* and *TNL60*, in this *TN* gene cluster (Figure 1), suggesting that the function of *TN4-TN12* genes in plant immunity may be related to *TNL40* and *TNL60*. Furthermore, *TNL40* and *TNL60* formed a head-to-head genomic arrangement with *TN4-TN12* (Figure 1), which was reminiscent of the head-to-head arrangement of *TNL* gene *SOC3* and *TN* genes *TN1* and *TN2* in the genome [31]. As both the TN1 and TN2 paired with SOC3 to monitor the homeostasis of SAUL1 [32,33], we hypothesized that TNL40 and TNL60 may also employ these TN proteins to monitor different substrates and activate downstream immune responses.

Exons and introns are represented by black boxes and solid lines, respectively. Arrows indicate the orientation of genes. The long bar at the bottom represents *Arabidopsis* chromosome 1. The gray dot represents the position of the centromere and the red dot represents the location of the indicated *TN* gene cluster. The red bars and numbers represent the boundaries of these *TN* gene cluster.

### 2.2. TN10 Interacts with TNL40 and TNL60

To investigate the functional link between these TNs and two TNL proteins, we first tested their interaction by performing firefly split-luciferase complementation (LUC) assays. As the activation and over-accumulation of TNL proteins may be toxic to plants, we generated a series of truncations of TNL40 and TNL60 based on their protein domains, including TNL40TIR, TNL40NBS, TNL40LRR, TNL60TIR, TNL60NBS, and TNL60LRR, respectively. In this LUC assay, the MYC-tagged N terminus of firefly luciferase (Nluc) was fused to the truncations of TNL40 and TNL60. Moreover, the C terminus (Cluc) was fused to each TN, except TN6 whose full-length coding sequence (CDS) was not cloned from *Arabidopsis* leaves, respectively. Different pairs of constructs were transiently expressed in 4-week-old *N. benthamiana* leaves and the luminescence signals were observed two days post inoculation (dpi). As shown in Supplementary Figure S1, we observed strong luminescence signals in both leaves co-expressing TN10 with TNL40TIR or TNL60TIR. We also confirmed the correct expression of these fusion proteins in the *N. benthamiana* leaves (Supplementary Figure S2), suggesting that TN10 may be the unique TN protein in this cluster that associated with both TNL40 and TNL60.

The TIR domain of the TNL protein is an intracellular signaling domain that triggers immunity, and the homo- or hetero-dimerization of TIR domains is crucial for immune signaling transduction [17,18,34]. To further confirm the interaction between TNs and TNL40 or TNL60, we next fused the TIR domain of each TN protein to Cluc and performed LUC assays with TNL40TIR-Nluc and TNL60TIR-Nluc, respectively. As shown in Supplementary Figure S3, we only observed strong luminescence signals in leaves co-expressing TNL40TIR-Nluc or TNL60TIR-Nluc with TN10TIR-Cluc, but not with other TNs Cluc-tagged TIR proteins, indicating that TNL40 and TNL60 mainly interacts with TN10. Therefore, we focused on TN10 mainly in the further investigation.

**Figure 2:**
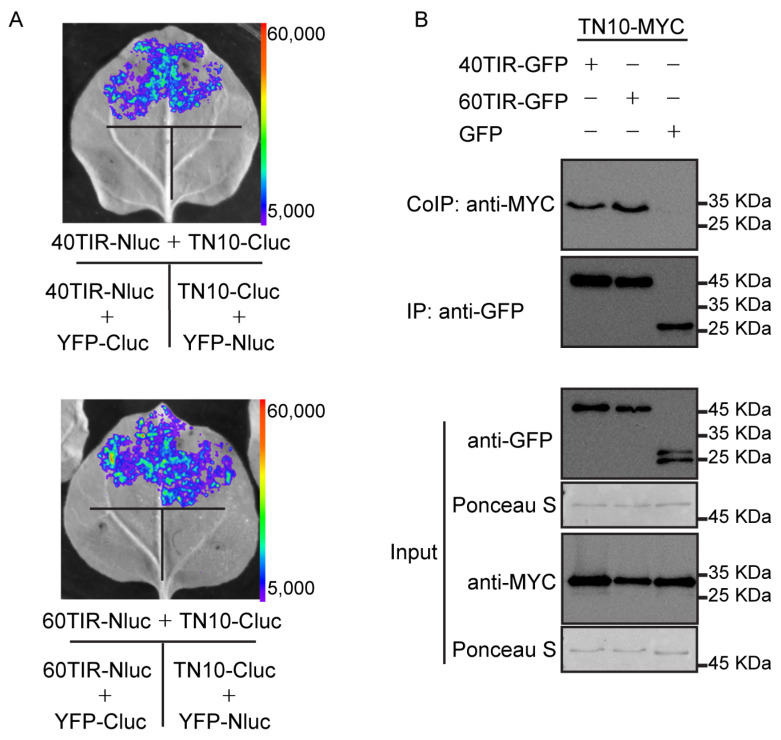
The Toll/interleukin-1 receptor (TIR) domains of TNL40 and TNL60 interact with TN10 *in planta*. (**A**) Images of *N. benthamiana* leaves two days after inoculation with indicated constructs in a firefly split-luciferase complementation imaging (LUC) assay. The coding sequence (CDS) of *TN10* was fused to the C-terminal fragment to firefly luciferase (*Cluc*) and the CDS of *TNL40TIR* or *TNL60TIR* was fused to the N-terminal fragment to firefly luciferase (*Nluc*). The indicated constructs were transiently co-expressed in 4-week-old *N. benthamiana* leaves. Yellow fluorescence protein (YFP)-Cluc and YFP-Nluc were used as the negative controls. (**B**) The interaction analysis of TNL40TIR-GFP or TNL60TIR-GFP with TN10-MYC in a co-immunoprecipitation (Co-IP) assay 16 h after transfection of *Arabidopsis* Col-0 protoplasts. The expression of GFP with TN10-MYC was used as a negative control. Proteins were detected by immunoblotting using anti-GFP and anti-MYC antibodies.

To confirm the interaction between TN10 and TNL40TIR or TNL60TIR, we next performed the LUC assay with appropriate controls. In this assay, we fused yellow fluorescence protein (YFP) to Cluc and Nluc, respectively, to act as negative controls. After co-expression of different pairs in *N. benthamiana* leaves, we found that TN10-Cluc associated with both TNL40TIR-Nluc and TNL60TIR-Nluc, but not with YFP-Nluc, and there is no interaction between YFP-Cluc and TNL40TIR-Nluc or TNL60TIR-Nluc (Figure 2A). We also confirmed the correct expression of all these fusion proteins (Supplementary Figure S4). These data indicate that TN10-Cluc associates with TNL40TIR-NLuc and TNL60TIR-Nluc specifically.

To further confirm this result, we next performed a co-immunoprecipitation (Co-IP) assay in which we transiently expressed GFP, TNL40TIR-GFP, or TNL60TIR-GFP with TN10-MYC in 4-week-old *Arabidopsis* protoplasts (Figure 2B). When we immunoprecipitated GFP, TNL40TIR-GFP, and TNL60TIR-GFP proteins using an anti-GFP antibody, TN10-MYC protein was detected with an anti-MYC antibody only in the precipitate from the leaves that co-expressed both TN10-MYC and TNL40TIR-GFP or TNL60TIR-GFP, but not from the negative control leaves that expressed TN10-MYC and GFP (Figure 2B), indicating again that TN10 can form a complex with TNL40TIR or TNL60TIR in *Arabidopsis*.

### 2.3. TN10 Contains A Typical TIR Domain

To gain more insights into the TN10 protein, we performed the alignments of protein amino acid sequences with the well-characterized TNL protein L6 and RESISTANCE TO PSEUDOMONAS SYRINGAE 4 (RPS4), as well as TN2. We found that all TN10, TNL40, and TNL60 contained a typical TIR domain (Supplementary Figure S5), including four conserved TIR subdomains (TIR1-TIR4) [30,36]. Several recent studies demonstrated that the TIR domains of TNL proteins were able to cleave nicotinamide adenine dinucleotide in its oxidized form (NAD^+^) and transmit signals downstream to trigger cell death [41,42,43]. Furthermore, a conserved glutamic acid (Glu, E) was found to be required for the activity of plant TIR domain to act as a NAD^+^-cleaving enzyme (NADase) [41]. As shown in Supplementary Figure S5, we also found that the conserved Glu was present in the TIR2 subdomain of all aligned TIR-type proteins, indicating again that TN10, TNL40, and TNL60 carry typical TIR domains.

Moreover, we found that the motifs defining the NBS, ARC1 (Apaf-1, R proteins, CED-4, 1), and ARC2 subdomains were conserved in TNL40, TNL60, L6, and RPS4 (Supplementary Figure S5) [44]. As typical TNL proteins, TNL40 and TNL60 also contained GKT-type Walker A/P-loop motifs, a conserved GNNNNGKT sequence (N represents any amino acid residue) that is essential for the binding of ATP [45,46] in their NBS domain, while it is a GRS-type P-loop motif (GNNNNGRS) in TN2 (Supplementary Figure S5). In addition, the conserved MHD (methionine-histidine-aspartate) motif required for function/activity of TNL was also observed in the NBS domains of TNL40 and TNL60 [44,47], but not in the NBS domains of TN2 and TN10 (Supplementary Figure S5), indicating again that TNL40 and TNL60 are typical TNL proteins. Different from other TN proteins, we found that TN10 not only lacks LRR domains, but also misses most of the NBS domain sequences, including the P-loop motif in its NBS domain (Supplementary Figure S5), suggesting that the function of TN10 in plant immunity may be different from other TN proteins.

### 2.4. Subcellular Localization of TNL40, TNL60, and TN10

**Figure 3:**
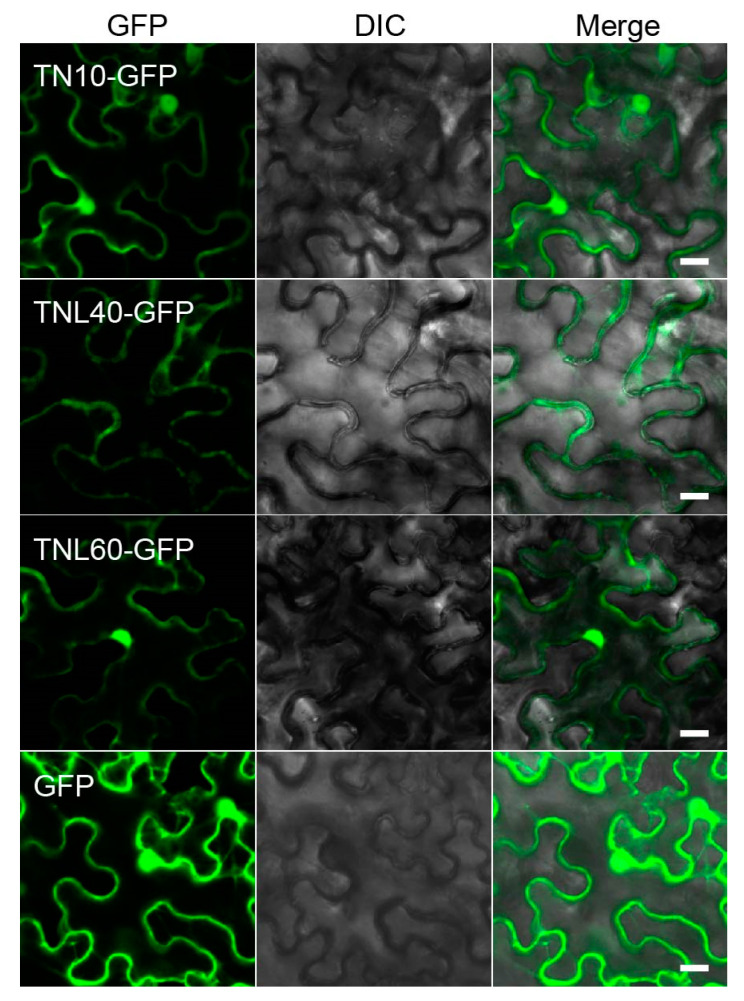
Subcellular localizations of TN10, TNL40, and TNL60. Subcellular localization of TN10, TNL40, and TNL60 in *N. benthamiana* leaves. The TN10-GFP, TNL40-GFP, and TNL60-GFP were transiently expressed in 4-week-old *N.benthamiana* leaves. GFP fluorescence was visualized 48 h after transformation. The GFP alone protein was used as a control. Bar = 20 μm.

In order to explore the function of TNL40, TNL60, and TN10, we next examined TNL40-GFP, TNL60-GFP, and TN10-GFP subcellular localization in *N. benthamiana* leaves. The expression of GFP alone was used as a negative control. Consistent with the previous report [22], we observed that TN10-GFP localized in both the cytoplasm and nucleus (Figure 3). Interestingly, TNL40 mainly localized in the cytoplasm (Figure 3 and Supplementary Figure S6), while TNL60 localized in the cytoplasm and nucleus (Figure 3). All proteins of the correct size without free GFP were detected by an immunoblotting analysis with an anti-GFP antibody (Supplementary Figure S7).

### 2.5. Overexpression of TNL40TIR and TNL60TIR Induced Cell Death in N. tabacum

**Figure 4:**
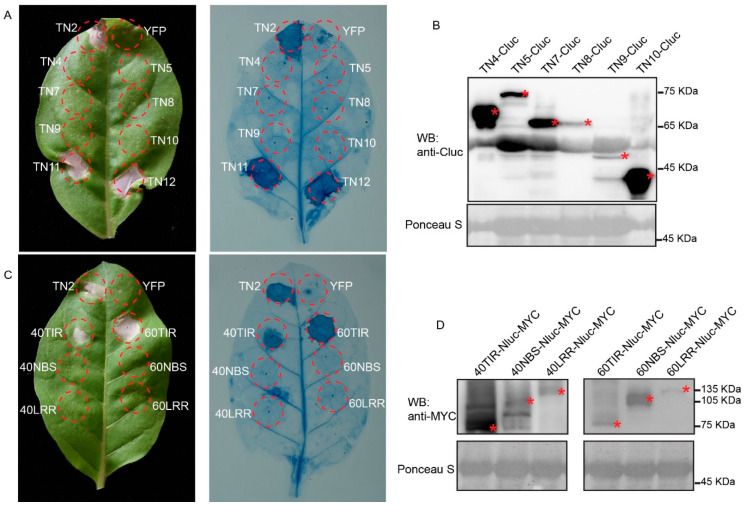
Overexpression of *Arabidopsis* TN4-TN12 and TNL40/60 canonical domain in *N. tabacum* leaves. (**A**) Transient overexpression of TN4-Cluc, TN5-Cluc, TN7-Cluc, TN8-Cluc, TN9-Cluc, TN10-Cluc, TN11-Cluc, and TN12-Cluc in *N. tabacum* leaves and cell death was observed and photographed at 4 days after injection. TN2 was used as a positive control and YFP-Cluc was used as a negative control. (**B**) Immunoblotting analysis of the accumulation of TN4-Cluc, TN5-Cluc, TN7-Cluc, TN8-Cluc, TN9-Cluc, TN10-Cluc proteins in *N. tabacum* leaves. Asterisks indicate the position of the corresponding proteins. (**C**) Transient overexpression of 40TIR-Nluc-MYC, 40NBS-Nluc-MYC, 40LRR-Nluc-MYC, 60TIR-Nluc-MYC, 60NBS-Nluc-MYC, 60LRR-Nluc-MYC in *N. tabacum* leaves and cell death was observed and photographed at 4 days after injection. TN2 was used as a positive control and YFP-Nluc was used as a negative control. (**D**) Immunoblotting analysis of the accumulation of 40TIR-Nluc-MYC, 40NBS-Nluc-MYC, 40LRR-Nluc-MYC, 60TIR-Nluc-MYC, 60NBS-Nluc-MYC, 60LRR-Nluc-MYC proteins in *N. tabacum* leaves. Asterisks (*) indicate the position of the corresponding proteins.

Overexpression of TNL proteins or their TIR domains in *N. tabacum* leaves often leads to cell death [18,41]. However, whether these TN proteins can trigger cell death is unclear. Then, with TN2 as a positive control, we transiently expressed TN4, TN5, TN7, TN8, TN9, TN10, TN11, and TN12 protein in *N. tabacum* leaves. Four days after injection, we observed that the expression of TN2 induced significant cell death, which is consistent with a previous report [26]. Similarly, we found that TN11 and TN12, but not other TNs in this cluster, can also trigger obvious cell death (Figure 4A), and the TN4, TN5, TN7, TN8, TN9, and TN10 proteins were accumulated at the correct size (Figure 4B). In addition, we also observed that the expression of the TIR domains, but not the NBS or LRR domains, of TNL40 and TNL60 can trigger cell death in *N. tabacum* leaves (Figure 4C,D).

**Figure 5:**
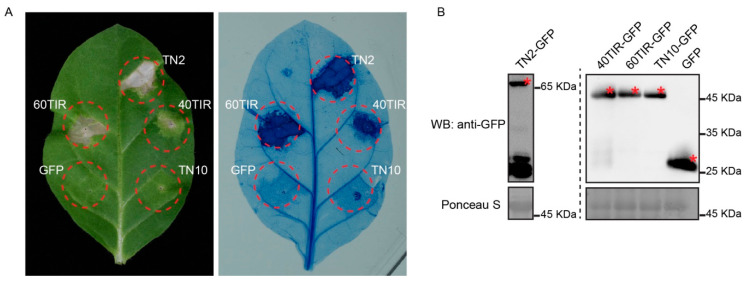
Transient overexpression of TNL40TIR and TNL60TIR in tobacco results in cell death. (**A**) Transient overexpression of TNL40TIR, TNL60TIR, and TN10 in *N. tabacum* leaves and the cell death were observed and photographed at 3 days after injection. TN2-GFP was used as a positive control and GFP was used as a negative control. (**B**) Immunoblotting analysis of the accumulation of TN2-GFP, TNL40TIR-GFP, TNL60TIR-GFP, TN10-GFP, and GFP proteins in *N. tabacum* leaves before cell death occurred. Asterisks indicate the position of the corresponding proteins.

Interestingly, we found that the cell death triggered by TNL40TIR was weaker than that by TNL60TIR. To further test this, we next expressed TNL40TIR-GFP, TNL60TIR-GFP, and TN10-GFP in *N. tabacum* leaves and observed the occurrence of cell death. As shown in Figure 5A, we found that the TNL60TIR triggered cell death was obvious 3 days after injection, while the TNL40TIR induced cell death was still not very obvious, indicating that the hypersensitive response activated by TNL40 was weaker than TNL60 (Figure 5A). Additionally, we also tested the accumulation of protein levels of TNL40TIR, TNL60TIR, and TN10, and found all proteins were expressed and accumulated (Figure 5B), indicating again that TNL40 and TNL60 carry typical and functional TIR domains.

### 2.6. Expression of TNL40, TNL60, and TN10 after Infected with Bacterial Pathogens

**Figure 6:**
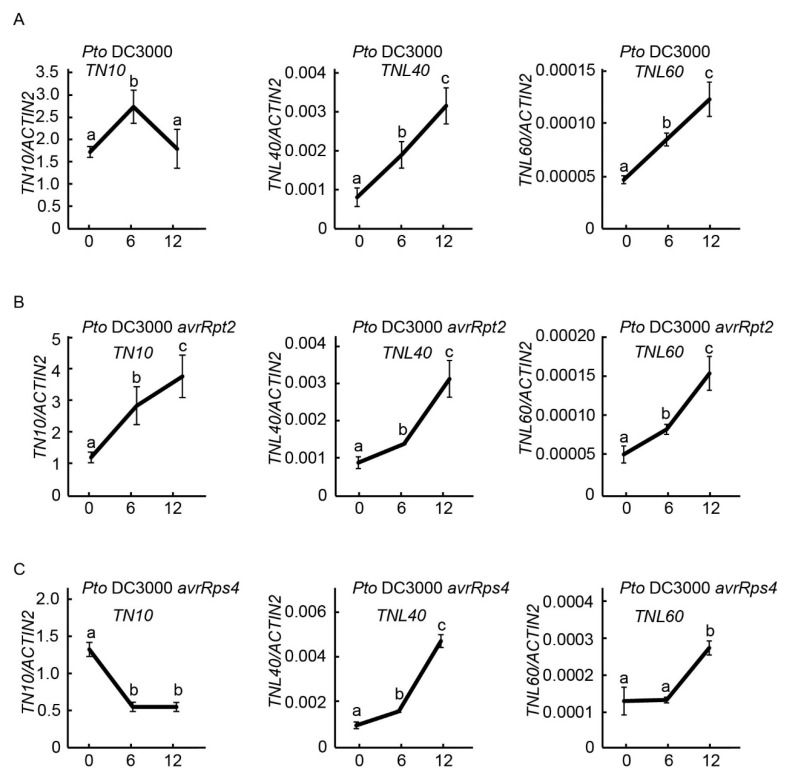
Expression of *TN10*, *TNL40*, and *TNL60* genes in *Arabidopsis* Col-0 plants after infiltration with different *Pto* DC3000 strains. The expression levels of *TN10*, *TNL40*, and *TNL60* genes were measured using qRT-PCR after inoculation with *Pto* DC3000 (**A**), *Pto* DC3000 *avrRpt2* (**B**), and *Pto* DC3000 *avrRps4* (**C**). The y-axis represents the relative expression level that was normalized to those of *ACTIN2*, and the x-axis represents the indicated time point (hours) after inoculation. Lower-case letters indicated statistically significant differences (*p* < 0.05, ANOVA, *n* = 3).

The pattern of expression of *TNL40, TNL60*, and *TN10* under inoculation with pathogens may provide some clues to their cellular roles. In order to explore the role and functional link of *TNL40*, *TNL60,* and *TN10* in disease resistance, we next inoculated the 4-week-old *Arabidopsis* wild-type Col-0 leaves with *Pto* DC3000, *Pto* DC3000 *avrRpt2*, and *Pto* DC3000 *avrRps4*, respectively, and analyzed the mRNA expression levels of *TNL40*, *TNL60*, and *TN10* by quantitative reverse transcription (qRT)-PCR. The initial transcription level of *TN10* was very high (Figure 6), which is consistent with the previous reports [22,23]. We found that both *TNL* genes, *TNL40* and *TNL60*, were up-regulated after being infected with *Pto* DC3000, *Pto* DC3000 *avrRpt2*, and *Pto* DC3000 *avrRps4* (Figure 6), suggesting that TNL40 and TNL60 may play a role after pathogens invasion. Interestingly, we found that the transcripts of *TN10* increased significantly six hours after treatment with *Pto* DC3000 and *Pto* DC3000 *avrRpt2*, but decreased significantly six hours after treatment with *Pto* DC3000 *avrRps4* (Figure 6), indicating that TN10 plays different roles in the resistance response mediated by different effectors. However, different to the inoculation with *Pto* DC3000 *avrRpt2*, we observed that the up-regulated expression of *TN10* was dropped to initial level 12 h after inoculation with *Pto* DC3000 (Figure 6A,B), suggesting that TN10 may only play a role in the resistance to *Pto* DC3000 at the early time of infection.

## 3. Discussion

The head-to-head arranged *NLR* gene pairs have been reported in many studies [33,38,40,48,49,50,51,52]. In this article, we found that there was a large *TN*-*TNL* gene cluster on *Arabidopsis* chromosome 1 and showed the association of TN10 with TNL40TIR and TNL60TIR. As far as we know, this is the first report that an atypical TN protein was shown to interact with the TIR domain of two full-length TNL proteins. Furthermore, we tested the subcellular localization of TNL40, TNL60, and TN10, and showed that TNL40 mainly localized in the cytoplasm, while TN10 and TNL60 localized in both the cytoplasm and nucleus. In addition, the observation of co-regulation of transcripts of *TN10* with *TNL40* and *TNL60* further indicated that TN10 may form complexes with TNL40 and TNL60 to regulate plant immunity.

The head-to-head genomic arrangements play important roles in regulating the cooperation of NLR proteins as almost all of the identified paired NLRs are encoded by physically linked genes [40]. For example, the SOC3-CHS1 pair and SOC3-TN2 pair are the two firstly identified unique NLR pairs formed by a full-length TNL and a truncated TN protein [33,49]. The over-accumulation and disappearance of *Arabidopsis* E3 ligase SAUL1 are monitored by SOC3-TN2 and SOC3-TN1, respectively [32,33]. *SOC3*, *CHS1*, and *TN2* genes also show head-to-head genomic arrangement on chromosome 1 [31,33,49]. These reports suggested that the analysis of the interaction of NLR proteins in gene clusters may reveal new NLR protein pairs [35,38,39,40,51]. We next analyzed the *Arabidopsis* genome and found that the largest *TNs* gene cluster was located on chromosome 1. Two typical *TNL* genes were found in this cluster, suggesting that they may function together in plant immunity. Interestingly, in our further investigation, we found that only TN10 in this cluster interacted strongly with both TIR domains of TNL40 and TNL60, indicating that TN10 may transmit signals to TNL40 and TNL60 to regulate plant immunity. Why other TNs in this cluster did not obviously interact with TNL40 or TNL60 and what targets were guarded by TNL40-TN10 and TNL60-TN10 pairs needs to be further investigated.

In plants, the TIR domain of TNL plays a crucial role in immune signaling since ectopic expression of certain TIR domain triggers strong cell death in tobacco [31,53]. Recent studies showed that, similar to the TIR domain of animals, the plant TIR domain also has NADase activity, which is required for cell death induction [41,42]. As shown in Supplementary Figure S5, we found that both the TIR domains of TNL40 and TNL60 contained the conserved glutamic acid, which is necessary for their potential NADase activity, in the putative catalytic residue. Consistent with this, both TNL40TIR and TNL60TIR could trigger cell death in *N. tabacum* leaves. Interestingly, although the alignments of protein sequences showed that TN10 contained the typical TIR domain and the conserved glutamic acid in its putative catalytic residue, we did not observe obvious cell death in *N. tabacum* leaves expressing TN10 in our experimental conditions. However, the TN10 protein was accumulated in *N. tabacum* leaves. Moreover, it is noteworthy that we found very high transcripts of *TN10* in the untreated plants, which is consistent with a previous report [22,23]. Together with the fact that TN10 protein misses most of the NBS domain sequences, including P-loop and MHD motifs, we proposed that TN10 may mainly function in transmitting immune signals. Furthermore, it is worth noting that the well-characterized TIR-only functional immune receptor RESPONSE TO HOPBA 1 (RBA1), which is sufficient to trigger cell death in response to HopBA1, functions as a pathogen sensor [54]. It is possible that TN10, as a TIR-only receptor, may be functionally similar to RBA1 in effector recognition and immune activation.

Similar to the pentamerized resistosome formed by the CNL protein ZAR1 [19,55], two more recent studies showed that the TNL proteins RPP1 and ROQ1 can form the tetramerized resistosome required for their NADase activity [56,57]. The existence of TNL resistosome may serve as a platform to recruit TIR-only proteins or other oligomerized TNLs to form complexes and expand TNL-mediated immune signals [56]. Similarly, whether TNL40 and TNL60 can form tetramerized resistosome remains to be determined. It is possible that TN10 may also participate in the process of TNL tetramerization resistosome. The prominent TIR domain in the resistosome enhances its NADase enzyme activity, so it will be interesting to determine whether TN10 affects the prominent TIR domain in the TNL resistosome. In addition, whether TN10 can form a tetramerization by its TIR domain is also an interesting question to be determined.

The mRNA expression levels of *TN10*, *TNL40*, and *TNL60* were co-regulated after inoculation with *Pto* DC3000 and *Pto* DC3000 *avrRpt2*, but not with *Pto* DC3000 *avrRps4*, suggesting that TN10 may function together with TNL40 and TNL60 in response to different effectors. Interestingly, we found that the up-regulation of *TN10* transcripts significantly occurred six hours after injection with *Pto* DC3000 and *Pto* DC3000 *avrRpt2*, but the expression of *TNL40* and *TNL60* was obviously increased 12 h after injection with *Pto* DC3000 and *Pto* DC3000 *avrRpt2*, indicating that the up-regulation of *TNL40* and *TNL60* is later than *TN10*, and TN10 may sense the attack of bacteria and transmit the signals to TNL40 and TNL60 to activate immunity. The expression trend of *TN10* after inoculation with *Pto* DC3000 is similar to the treatment of salicylic acid (SA) [22], indicating that TN10 plays a positive role in the early stage of immune responses, which is consistent with the previous report that the TN10 overexpression plants display enhanced resistance to *Pto* DC3000 [22]. However, it will also be interesting to investigate why the transcripts of *TN10* are induced by *Pto* DC3000 *avrRpt2* as the effector AvrRpt2 is recognized by the well-characterized CNL RPS2.

**Figure 7:**
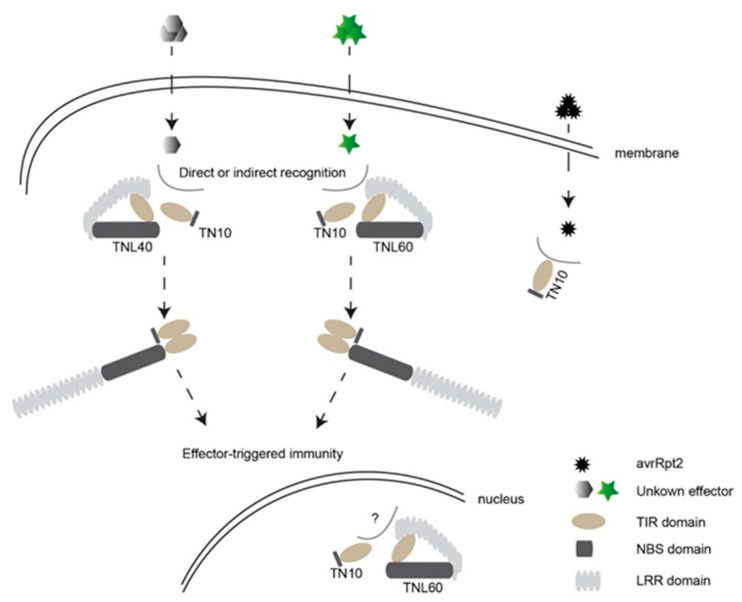
A possible model summarizing the function of TNL40, TNL60, and TN10 proteins in plant immunity. In the resting cell, TNL40 and TNL60 are kept in inactive states through the interaction between their domains. When plants were attacked by pathogens, TN10 interacted with TNL40 or TNL60 and resulted in the changes of the conformation of TNL proteins followed by the activation of effector-triggered immunity (ETI) responses. TNL40, TNL60, and TN10 may also function as accessory components in signal transduction during defense responses.

*Arabidopsis* ecotype Col-0 has only about 82 TNLs and 51 CNLs, which are sufficient to deal with the invasion and colonization of most external pathogens [23]. How plants use limited NLR proteins to resist the complex environment has become a key issue. Previous studies have suggested that the proteins encoded by the clustered *NLRs* may act together in activating immune responses and may be likely to provide coregulatory benefits [40]. Our results here showed that the atypical TN protein TN10 can interact with TNL40TIR and TNL60TIR. Based on these results, we proposed a possible working model for the functional mechanism of TN10, TNL40, and TNL60 in plant immunity (Figure 7). In the resting cell, TNL40 and TNL60 are kept in inactive states through the interaction between their domains. When pathogens such as bacteria secrete effectors into plant cells, TN10 interacts with the TIR domain of TNL40 or TNL60, leading to the change of the conformation of the full-length TNL proteins, thereby releasing the full-length TNL to activate ETI responses. Additionally, how TN10 activates and helps the full-length TNL proteins perform functions are also interesting questions that need to be determined in the further investigation.

In summary, our results here showed that *TNL40*, *TNL60*, and *TN10* were located in the largest *TN-TNL* gene cluster in a head-to-head genomic arrangement. Transient expression of the TIR domains of TNL40 and TNL60 triggered cell death in *N. tabacum* leaves. Moreover, TN10 interacted with both TNL40TIR and TNL60TIR, and the observation of co-expression of *TNL40*, *TNL60*, and *TN10* after infection with *Pto* DC3000 suggested that TN10 pairs with TNL40 and TNL60 may contribute to immune signaling.

## 4. Materials and Methods

### 4.1. Plant Materials and Growth Conditions

The wild-type *Arabidopsis thaliana* accession used in this study was Columbia-0 (Col-0), seeds were surface-sterilized by 70% ethanol and planted on plates containing a half-strength Murashige and Skoog (1/2 MS) medium with 1% sucrose. Plates were kept in 4 °C for 3 days and moved to the growth room, and then transplanted into soil (nutrient soil/vermiculite 2: 1) 7 days after germination. *Arabidopsis thaliana* plants were grown in a growth room at 22 °C with approximately 60% relative humidity and 9-h-light/15-h-dark photoperiod, with a light intensity of 7000–8000 lux for protoplasts preparation and gene expression assay. *Nicotiana benthamiana* and *Nicotiana tabacum* plants were grown under the same growth conditions as *Arabidopsis* [58].

### 4.2. Plasmid Constructs

All CDS sequences containing *TN4*-*TN12*, *TNL40,* and *TNL60* used in this research were derived from the *Arabidopsis thaliana* Col-0 accession. Plasmids used in this study were constructed by In-fusion technology’s One-Step Cloning Kit (Vazyme, Nanjing, China. C112). All clones were verified with DNA sequencing. For the firefly split-luciferase complementation assay, the CDS of *TN4*, *TN5*, *TN7*, *TN8*, *TN9*, *TN10*, *TN11*, *TN12*, *TN4TIR*, *TN5TIR*, *TN7TIR*, *TN8TIR*, *TN9TIR*, *TN11TIR*, and *TN12TIR* were amplified with specific primers and then cloned into pCAMBIA1300-Cluc, respectively. The CDS of *TNL40TIR*, *TNL40NBS*, *TNL40LRR*, *TNL60TIR*, *TNL60NBS*, and *TNL60LRR* were amplified with specific primers and then cloned into pCAMBIA1300-Nluc-MYC, respectively. For the Co-IP assay, *TNL40TIR* and *TNL60TIR* was cloned into pSuper1300 with a GFP tag, *TN10* was cloned into pSuper1300 with a 6 × MYC tag. For the subcellular localization assay, the full-length *TNL40*, *TNL60* and *TN10* CDS sequences were amplified and then cloned into pSuper1300 with a GFP tag. The primers used for the plasmid constructs are listed in Supplementary Table S1.

### 4.3. Cell Death Assay in N. tabacum Leaves and Tobacco Protein Extraction

*Agrobacterium* strain GV3101 carrying the targeted plasmid was resuspended to OD _600_ = 0.4 in an injection buffer (10 mM MES [pH 5.6], 10 mM MgCl_2_, 150 μM Acetosyringone), then gently injected into the tobacco leaf using a 1-mL needle-free syringe. The tobacco was cultivated in the dark for 12 h, then transferred to the short-day growth conditions. For cell death leaf observation experiments, the pictures were taken at 3 or 4 days after injection. Then, the indicated *N. tabacum* leaves were stained with trypan blue and decolorized overnight. For protein extraction experiments, the samples were taken 36 h after injection (before cell death occurrence). The leaves were frozen quickly in liquid nitrogen and ground into powders in a mortar. Then, the sample was transferred to a new 2-mL centrifuge tube. Native extraction buffer (50 mM Tris-MES [pH 8.0], 0.5 M Sucrose, 1 mM MgCl_2_, 10 mM EDTA, 5 mM DTT, and 1% [*w*/*v*] protease inhibitor cocktail S8830 [Sigma, St. Louis, MO, USA]) was added in a ratio of 1 g to 2 mL. The tubes were placed on ice for 30 min and centrifuged at 12,000 *g* for 15 min, and then the supernatant was transferred to a new centrifuge tube to be used in the next protein immunoblotting assay performed as described previously [59].

### 4.4. Firefly Split-Luciferase Complementation Assay

Pairs of *Agrobacterium* GV3101 strains containing the targeted plasmid combinations were resuspended in an injection buffer (the final concentration of each stain injection was OD _600_ = 0.4), and then co-transformed into 4-week-old *N. benthamiana* leaves and incubated in the short-day condition growth room for 48 h. The *N. benthamiana* leaves were sprayed with 1 mM precooled luciferin, and the samples were placed in darkness for 5 min. Subsequently, LUC images were captured with a low-light cooled CCD imaging apparatus. The details of this assay were described previously [60].

### 4.5. Transient Expression in Arabidopsis Protoplasts

Protoplasts were prepared from 4-week-old *Arabidopsis* Col-0 leaves and transfections were done as described previously [61]. After transfection, protoplasts were incubated at room temperature under weak light for 16 h, and protein extracts were prepared for immunoblotting and Co-IP assays.

### 4.6. Protoplast Protein Extraction, Immunoprecipitation, and Immunoblotting

Protoplast proteins were processed in extraction buffer (50 mM Tris [pH 7.5], 150 mM NaCl, 10% (*v*/*v*) glycerol, 2 mM EDTA, 5 mM DTT, 1% [*w*/*v*] protease inhibitor cocktail S8830 [Sigma], 0.1% Triton). Lysates were centrifuged for 15 min at 14,000 rpm at 4 °C. Aliquots of supernatants were used as input samples. For immunoprecipitation assays, 20 μL GFP-Trap Magnetic Agarose (Chromotek, Munich, Germany) was added to 1 mL total protein extract for 2 h incubation at 4 °C. Beads were collected by centrifugation and washed four times with a PBS buffer containing 0.1% (*v*/*v*) Triton X-100. Then, beads were resuspended in 80 μL of PBS buffer with SDS loading buffer and incubated at 95 °C for 8 min. Proteins were separated via SDS-PAGE gels and analyzed by immunoblotting. The anti-bodies used were anti-GFP (Sigma Aldrich, Louis, MO, USA, 11814460001) and anti-MYC (Sigma Aldrich, Louis, MO, USA, 11867423001).

### 4.7. Subcellular Localization Assay

The constructs were transformed into the *Agrobacterium* GV3101 strain and injected into *N. benthamiana* leaves. Plants were placed in the dark for 12 h and then transferred to the short-day growth conditions for 36 h, then the leaves were taken for observation. Green fluorescent protein (GFP) fluorescence was visualized using a confocal laser scanning microscope (Zeiss 880) under a × 20 objective, zoom × 0.8, 2048 × 2048 pixels, and imaged TN10-GFP and TNL40/60-GFP with 2 and 10% of the 488-nm line, respectively, and detected in the range 494–598 nm.

### 4.8. Pathogen Infection Assays and Gene Expression Analysis

*Pseudomonas syringae* pv. *tomato* (*Pto*) DC3000 or *Pto* DC3000 *AvrRpt2*, *Pto* DC3000 *AvrRps4* strains were cultured on the King’ B medium containing appropriate antibiotics at 28 °C for two days. The 10 mM MgCl_2_ containing buffer was used to resuspend bacteria to OD_600_ = 0.1. Leaves from 4-week-old plants were infiltrated with suspensions of different *Pto* DC3000 strains. The samples were taken at 0, 6, and 12 h after inoculation and frozen in liquid nitrogen. Total RNA was extracted by the TRIzol reagent (Invitrogen, Carlsbad, CA, USA) according to the manufacturer’s instructions, and the first strand was synthesized using a RT reagent kit (code number is RR047A; Takara, Dalian, China). Accumulation of transcripts was examined by qRT-PCR using the RT reagent Kit (code number is RR047A; Takara, Dalian, China). All qRT-PCR assays were performed with the Premix Ex Taq Kit (code number is RR420A; TaKaRa, Dalian, China) in a CFX Connect Real-time PCR System (BIO-RAD, Hercules, CA, USA). The *ACTIN2* gene was used as an internal control. All of the primers used for qRT-PCR are listed in Supplementary Table S1.

## 5. Conclusions

In this study, we identified the largest *TN-TNL* gene cluster in the *Arabidopsis* ecotype Col-0 genome. Two full-length *TNL* genes, *TNL40* and *TNL60*, were arranged in a head-to-head orientation with *TN4*-*TN12*. TNL40 and TNL60 interacted strongly with TN10, which is almost a naked TIR domain. TNL40, TNL60, and TN10 localized in the cytoplasm, and TN10 and TNL60 also localized in the nucleus. The transcripts of *TNL40*, *TNL60*, and *TN10* were co-induced when inoculated with *Pto* DC3000. Furthermore, both TNL40TIR and TNL60TIR induced cell death in *N. tabacum* leaves. Based on these data, we hypothesized that TNL40 and TNL60 were kept in inactive states in the resting cell. When bacterial pathogens attacked, TN10 interacted with TNL40 or TNL60 to change the conformation of the full-length TNLs, and then activated the ETI immune signaling.

## Data Availability

Sequence data can be found in The *Arabidopsis* Information Resource or GenBank/EMBL databases under the following numbers: *TN2* (*At1g17615*), *TN4* (*At1g72850*), *TN5* (*At1g72870*), *TN6* (*At1g72890*), *TN7* (*At1g72900*), *TN8* (*At1g72910*), *TN9* (*At1g72920*), *TN10* (*At1g72930*), *TN11* (*At1g72940*), *TN12* (*At1g72950*), *TNL40* (*At1g72840*), *TNL60* (*At1g72860*), *RPS4* (*At5g45250*), *L6* (*AAA91021*).

## Supplementary Materials

The Supplementary Materials are available online at https://www.mdpi.com/article/10.3390/ijms22084004/s1.

## References

[1] Teixeira P.J.P., Colaianni N.R., Fitzpatrick C.R., Dangl J.L. (2019). Beyond pathogens: Microbiota interactions with the plant immune system. Curr. Opin. Microbiol..

[2] Jones J.D.G., Dangl J.L. (2006). The plant immune system. Nature.

[3] Dangl J.L., Horvath D.M., Staskawicz B.J. (2013). Pivoting the plant immune system from dissection to deployment. Science.

[4] Kourelis J., van der Hoorn R.A.L. (2018). Defended to the nines: 25 years of resistance gene cloning identifies nine mechanisms for R protein function. Plant Cell.

[5] Zhou J.M., Zhang Y. (2020). Plant immunity: Danger perception and signaling. Cell.

[6] Yu X., Feng B., He P., Shan L. (2017). From chaos to harmony: Responses and signaling upon microbial pattern recognition. Annu. Rev. Phytopathol..

[7] Tang D., Wang G., Zhou J.M. (2017). Receptor kinases in plant-pathogen interactions: More than pattern recognition. Plant Cell.

[8] Zhang R., Zheng F., Wei S., Zhang S., Li G., Cao P., Zhao S. (2019). Evolution of disease defense genes and their regulators in plants. Int. J. Mol. Sci..

[9] Gómez-Gómez L., Boller T. (2000). FLS2: An LRR receptor-like kinase involved in the perception of the bacterial elicitor flagellin in *Arabidopsis*. Mol. Cell.

[10] Bauer Z., Gómez-Gómez L., Boller T., Felix G. (2001). Sensitivity of different ecotypes and mutants of *Arabidopsis thaliana* toward the bacterial elicitor flagellin correlates with the presence of receptor-binding sites. J. Biol. Chem..

[11] Kunze G., Zipfel C., Robatzek S., Niehaus K., Boller T., Felix G. (2004). The N terminus of bacterial elongation factor Tu elicits innate immunity in *Arabidopsis* plants. Plant Cell.

[12] Zipfel C., Kunze G., Chinchilla D., Caniard A., Jones J.D.G., Boller T., Felix G. (2006). Perception of the bacterial PAMP EF-Tu by the receptor EFR restricts *Agrobacterium*-mediated transformation. Cell.

[13] Cabeen M.T., Losick R. (2015). Bacterial backstabbing: EF-Tu, brute?. Cell.

[14] Wang W., Liu N., Gao C., Cai H., Romeis T., Tang D. (2020). The *Arabidopsis* exocyst subunits EXO70B1 and EXO70B2 regulate FLS2 homeostasis at the plasma membrane. New Phytol..

[15] Monteiro F., Nishimura M.T. (2018). Structural, functional, and genomic diversity of plant NLR proteins: An evolved resource for rational engineering of plant immunity. Annu. Rev. Phytopathol..

[16] Cesari S. (2018). Multiple strategies for pathogen perception by plant immune receptors. New Phytol..

[17] Cui H., Tsuda K., Parker J.E. (2015). Effector-triggered immunity: From pathogen perception to robust defense. Annu. Rev. Plant Biol..

[18] Wang W., Feng B., Zhou J.M., Tang D. (2020). Plant immune signaling: Advancing on two frontiers. J. Integr. Plant Biol..

[19] Wang J., Hu M., Wang J., Qi J., Han Z., Wang G., Qi Y., Wang H.W., Zhou J.M., Chai J. (2019). Reconstitution and structure of a plant NLR resistosome conferring immunity. Science.

[20] Maekawa T., Kufer T.A., Schulze-Lefert P. (2011). NLR functions in plant and animal immune systems: So far and yet so close. Nat. Immunol..

[21] Li X., Kapos P., Zhang Y. (2015). NLRs in plants. Curr. Opin. Immunol..

[22] Nandety R.S., Caplan J.L., Cavanaugh K., Perroud B., Wroblewski T., Michelmore R.W., Meyers B.C. (2013). The role of TIR-NBS and TIR-X proteins in plant basal defense responses. Plant Physiol..

[23] Meyers B.C., Morgante M., Michelmore R.W. (2002). TIR-X and TIR-NBS proteins: Two new families related to disease resistance TIR-NBS-LRR proteins encoded in *Arabidopsis* and other plant genomes. Plant J..

[24] Nasim Z., Fahim M., Gawarecka K., Susila H., Jin S., Youn G., Ahn J.H. (2020). Role of *AT1G72910*, *AT1G72940*, and *ADR1-LIKE 2* in plant immunity under nonsense-mediated mRNA decay-compromised conditions at low temperatures. Int. J. Mol. Sci..

[25] Zhao T., Rui L., Li J., Nishimura M.T., Vogel J.P., Liu N., Liu S., Zhao Y., Dangl J.L., Tang D. (2015). A truncated NLR protein, TIR-NBS2, is required for activated defense responses in the *exo70B1* mutant. PLoS Genet..

[26] Wang W., Liu N., Gao C., Rui L., Tang D. (2019). The *Pseudomonas syringae* effector AvrPtoB associates with and ubiquitinates *Arabidopsis* exocyst subunit EXO70B1. Front. Plant Sci..

[27] Liu N., Hake K., Wang W., Zhao T., Romeis T., Tang D. (2017). CALCIUM-DEPENDENT PROTEIN KINASE5 associates with the truncated NLR protein TIR-NBS2 to contribute to *exo70B1*-mediated immunity. Plant Cell.

[28] Roth C., Lüdke D., Klenke M., Quathamer A., Valerius O., Braus G.H., Wiermer M. (2017). The truncated NLR protein TIR-NBS13 is a MOS6/IMPORTIN-α3 interaction partner required for plant immunity. Plant J..

[29] Wang Y., Zhang Y., Wang Z., Zhang X., Yang S. (2013). A missense mutation in CHS1, a TIR-NB protein, induces chilling sensitivity in *Arabidopsis*. Plant J..

[30] Zbierzak A.M., Porfirova S., Griebel T., Melzer M., Parker J.E., Dörmann P. (2013). A TIR-NBS protein encoded by *Arabidopsis Chilling Sensitive 1* (*CHS1*) limits chloroplast damage and cell death at low temperature. Plant J..

[31] Zhang Y., Wang Y., Liu J., Ding Y., Wang S., Zhang X., Liu Y., Yang S. (2017). Temperature-dependent autoimmunity mediated by *chs1* requires its neighboring *TNL* gene *SOC3*. New Phytol..

[32] Tong M., Kotur T., Liang W., Vogelmann K., Kleine T., Leister D., Brieske C., Yang S., Lüdke D., Wiermer M. (2017). E3 ligase SAUL1 serves as a positive regulator of PAMP-triggered immunity and its homeostasis is monitored by immune receptor SOC3. New Phytol..

[33] Liang W., van Wersch S., Tong M., Li X. (2019). TIR-NB-LRR immune receptor SOC3 pairs with truncated TIR-NB protein CHS1 or TN2 to monitor the homeostasis of E3 ligase SAUL1. New Phytol..

[34] Williams S.J., Sohn K.H., Wan L., Bernoux M., Sarris P.F., Segonzac C., Ve T., Ma Y., Saucet S.B., Ericsson D.J. (2014). Structural basis for assembly and function of a heterodimeric plant immune receptor. Science.

[35] Huh S.U., Cevik V., Ding P., Duxbury Z., Ma Y., Tomlinson L., Sarris P.F., Jones J.D.G. (2017). Protein-protein interactions in the RPS4/RRS1 immune receptor complex. PLoS Pathog..

[36] Meyers B.C., Kozik A., Griego A., Kuang H., Michelmore R.W. (2003). Genome-wide analysis of NBS-LRR-encoding genes in *Arabidopsis*. Plant Cell.

[37] Xu F., Zhu C., Cevik V., Johnson K., Liu Y., Sohn K., Jones J.D., Holub E.B., Li X. (2015). Autoimmunity conferred by *chs3-2D* relies on *CSA1*, its adjacent TNL-encoding neighbour. Sci. Rep..

[38] Cesari S., Kanzaki H., Fujiwara T., Bernoux M., Chalvon V., Kawano Y., Shimamoto K., Dodds P., Terauchi R., Kroj T. (2014). The NB-LRR proteins RGA4 and RGA5 interact functionally and physically to confer disease resistance. EMBO J..

[39] Zhai C., Zhang Y., Yao N., Lin F., Liu Z., Dong Z., Wang L., Pan Q. (2014). Function and interaction of the coupled genes responsible for *Pik-h* encoded rice blast resistance. PLoS ONE.

[40] van Wersch S., Li X. (2019). Stronger when together: Clustering of plant *NLR* disease *resistance* genes. Trends Plant Sci..

[41] Wan L., Essuman K., Anderson R.G., Sasaki Y., Monteiro F., Chung E.H., Osborne Nishimura E., DiAntonio A., Milbrandt J., Dangl J.L. (2019). TIR domains of plant immune receptors are NAD^+^-cleaving enzymes that promote cell death. Science.

[42] Horsefield S., Burdett H., Zhang X., Manik M.K., Shi Y., Chen J., Qi T., Gilley J., Lai J.S., Rank M.X. (2019). NAD^+^ cleavage activity by animal and plant TIR domains in cell death pathways. Science.

[43] Chang M., Clinton M., Liu F., Fu Z.Q. (2019). NAD^+^ cleavage: TIR domain-containing resistance proteins in action. Trends Plant Sci..

[44] van Ooijen G., Mayr G., Kasiem M.M.A., Albrecht M., Cornelissen B.J.C., Takken F.L.W. (2008). Structure-function analysis of the NB-ARC domain of plant disease resistance proteins. J. Exp. Bot..

[45] Takken F.L.W., Albrecht M., Tameling W.I. (2006). Resistance proteins: Molecular switches of plant defence. Curr. Opin. Plant Biol..

[46] Bonardi V., Cherkis K., Nishimura M.T., Dangl J.L. (2012). A new eye on NLR proteins: Focused on clarity or diffused by complexity?. Curr. Opin. Immunol..

[47] Howles P., Lawrence G., Finnegan J., McFadden H., Ayliffe M., Dodds P., Ellis J. (2005). Autoactive alleles of the flax *L6* rust *resistance* gene induce non-race-specific rust resistance associated with the hypersensitive response. Mol. Plant Microbe Interact..

[48] Yang D., Li S., Lu L., Fang J., Wang W., Cui H., Tang D. (2020). Identification and application of the *Pigm-1* gene in rice disease-resistance breeding. Plant Biol..

[49] Liang W., Tong M., Li X. (2020). SUSA2 is an F-box protein required for autoimmunity mediated by paired NLRs SOC3-CHS1 and SOC3-TN2. Nat. Commun..

[50] Narusaka M., Iuchi S., Narusaka Y. (2017). Analyses of natural variation indicates that the absence of RPS4/RRS1 and amino acid change in RPS4 cause loss of their functions and resistance to pathogens. Plant Signal. Behav..

[51] Deng Y., Zhai K., Xie Z., Yang D., Zhu X., Liu J., Wang X., Qin P., Yang Y., Zhang G. (2017). Epigenetic regulation of antagonistic receptors confers rice blast resistance with yield balance. Science.

[52] Heidrich K., Tsuda K., Blanvillain-Baufumé S., Wirthmueller L., Bautor J., Parker J.E. (2013). *Arabidopsis* TNL-WRKY domain receptor RRS1 contributes to temperature-conditioned RPS4 auto-immunity. Front. Plant Sci..

[53] Ma Y., Guo H., Hu L., Martinez P.P., Moschou P.N., Cevik V., Ding P., Duxbury Z., Sarris P.F., Jones J.D.G. (2018). Distinct modes of derepression of an *Arabidopsis* immune receptor complex by two different bacterial effectors. Proc. Natl. Acad. Sci. USA.

[54] Nishimura M.T., Anderson R.G., Cherkis K.A., Law T.F., Liu Q.L., Machius M., Nimchuk Z.L., Yang L., Chung E.H., El Kasmi F. (2017). TIR-only protein RBA1 recognizes a pathogen effector to regulate cell death in *Arabidopsis*. Proc. Natl. Acad. Sci. USA.

[55] Feng B., Tang D. (2019). Mechanism of plant immune activation and signaling: Insight from the first solved plant resistosome structure. J. Integr. Plant Biol..

[56] Ma S., Lapin D., Liu L., Sun Y., Song W., Zhang X., Logemann E., Yu D., Wang J., Jirschitzka J. (2020). Direct pathogen-induced assembly of an NLR immune receptor complex to form a holoenzyme. Science.

[57] Martin R., Qi T., Zhang H., Liu F., King M., Toth C., Nogales E., Staskawicz B.J. (2020). Structure of the activated ROQ1 resistosome directly recognizing the pathogen effector XopQ. Science.

[58] Zhao Y., Wu G., Shi H., Tang D. (2019). RECEPTOR-LIKE KINASE 902 associates with and phosphorylates BRASSINOSTEROID-SIGNALING KINASE1 to regulate plant immunity. Mol. Plant.

[59] Gao C., Sun P., Wang W., Tang D. (2021). *Arabidopsis* E3 ligase KEG associates with and ubiquitinates MKK4 and MKK5 to regulate plant immunity. J. Integr. Plant Biol..

[60] Chen H., Zou Y., Shang Y., Lin H., Wang Y., Cai R., Tang X., Zhou J.M. (2008). Firefly luciferase complementation imaging assay for protein-protein interactions in plants. Plant Physiol..

[61] Yoo S.D., Cho Y.H., Sheen J. (2007). *Arabidopsis* mesophyll protoplasts: A versatile cell system for transient gene expression analysis. Nat. Protoc..

