# Supplementary material for: A Truncated TIR-NBS Protein TN10 Pairs with Two Clustered TIR-NBS-LRR Immune Receptors and Contributes to Plant Immunity in Arabidopsis
Source: Int J Mol Sci. 2021 Apr 13;22(8):4004. doi: 10.3390/ijms22084004 (PMC8069298; doi:10.3390/ijms22084004)
Supplement: Supplementary file 1 [file ijms-22-04004-s001.zip › Final supps/Supplementary Figure 1.pdf]

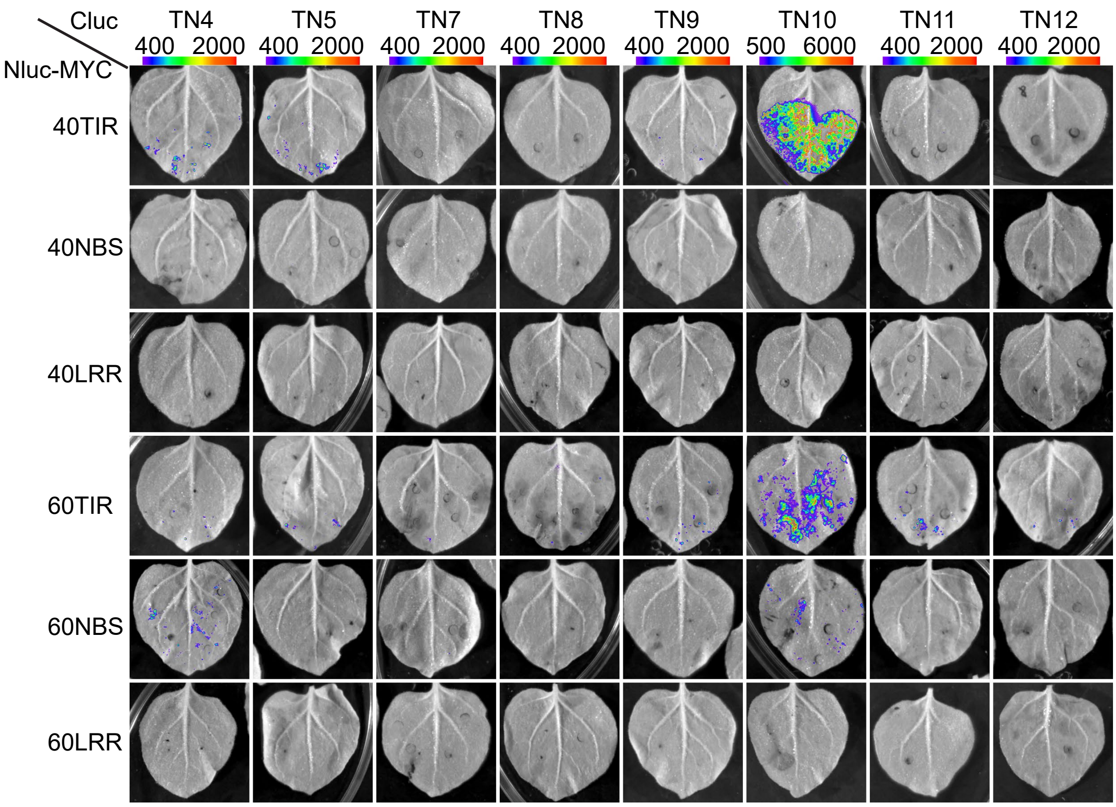

Supplementary Figure 1. Interaction screening of TNL40/60 TIR, NBS or LRR domain with TN4-TN12. The interaction between TN4-TN12 with TNL40/60 TIR, NBS or LRR domain was examined by performing the LUC assay in *N. benthamiana*. The CDS of *TN4*, *TN5*, *TN7*, *TN8*, *TN9*, *TN10*, *TN11* and *TN12* were fused to the *Cluc* and the CDS of *40TIR*, *40NBS*, *40LRR*, *60TIR*, *60NBS* and *60LRR* were fused to the *Nluc-MYC*. Different pairs of constructs were expressed in the *N. benthamiana* leaves by using indicated *Agrobacterium tumefaciens* strain GV3101. The infiltrated leaves were sprayed with 1 mM luciferin 48 hours after injection and the luminescence signal was captured by a cooled charge coupled device (CCD) camera.
