# Supplementary material for: A Truncated TIR-NBS Protein TN10 Pairs with Two Clustered TIR-NBS-LRR Immune Receptors and Contributes to Plant Immunity in Arabidopsis
Source: Int J Mol Sci. 2021 Apr 13;22(8):4004. doi: 10.3390/ijms22084004 (PMC8069298; doi:10.3390/ijms22084004)
Supplement: Supplementary file 1 [file ijms-22-04004-s001.zip › Final supps/Supplementary Figure 10.pdf]

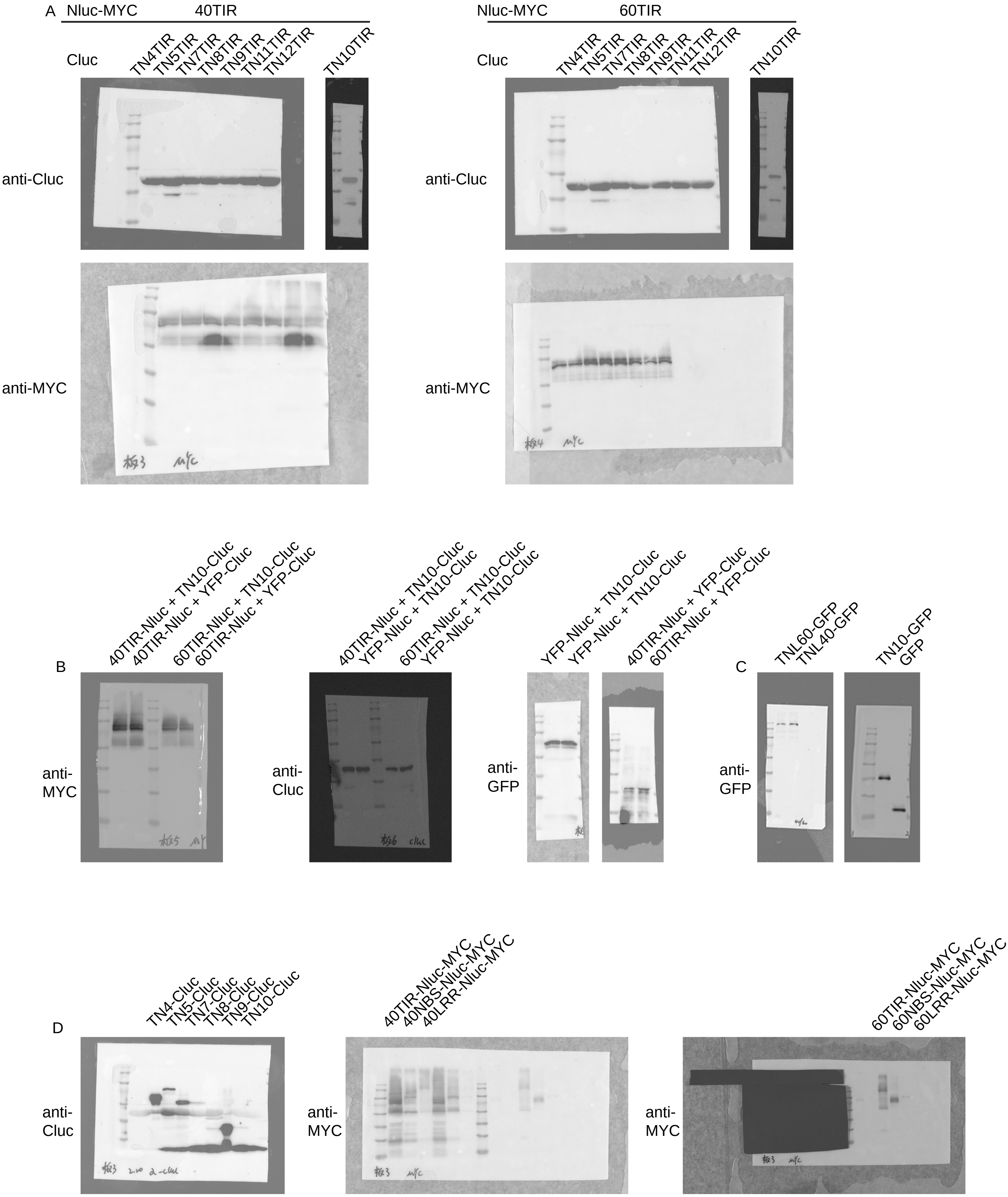

Supplementary Figure 10. The full size western blots gels of Supplementary Figure 3B, Supplementary Figure 4, Supplementary Figure 6, Supplementary Figure 7B and 7D

(A) The full size western blots gels of Supplementary Figure 3B

(B) The full size western blots gels of Supplementary Figure 4

(C) The full size western blots gels of Supplementary Figure 6

(D) The full size western blots gels of Supplementary Figure 7B and 7D
