# Supplementary material for: A Truncated TIR-NBS Protein TN10 Pairs with Two Clustered TIR-NBS-LRR Immune Receptors and Contributes to Plant Immunity in Arabidopsis
Source: Int J Mol Sci. 2021 Apr 13;22(8):4004. doi: 10.3390/ijms22084004 (PMC8069298; doi:10.3390/ijms22084004)
Supplement: Supplementary file 1 [file ijms-22-04004-s001.zip › Final supps/Supplementary Figure 2.pdf]

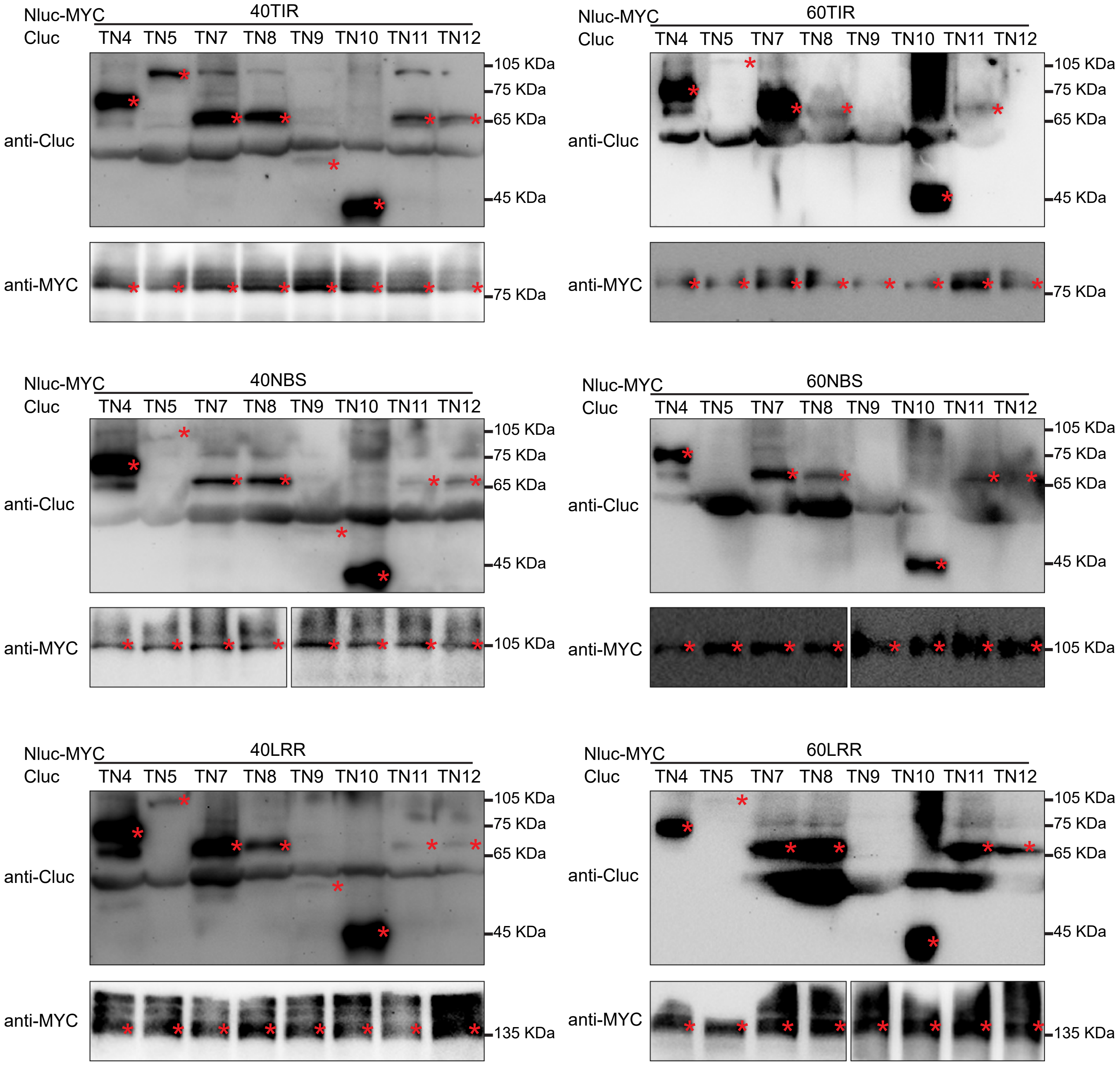

Supplementary Figure 2. Immunoblotting analysis of co-expression 40TIR, 40NBS, 40LRR, 60TIR, 60NBS, 60LRR with TN4-TN12 in *N. benthamiana*.

Different pairs of constructs were transiently expressed in *N. benthamiana* leaves as described in Supplementary Figure 1, the TN4-TN12 proteins were detected by anti-Cluc and 40TIR, 40NBS, 40LRR, 60TIR, 60NBS, 60LRR were detected by anti-MYC. Asterisks (*) indicate the position of the corresponding proteins.
