# Supplementary material for: A Truncated TIR-NBS Protein TN10 Pairs with Two Clustered TIR-NBS-LRR Immune Receptors and Contributes to Plant Immunity in Arabidopsis
Source: Int J Mol Sci. 2021 Apr 13;22(8):4004. doi: 10.3390/ijms22084004 (PMC8069298; doi:10.3390/ijms22084004)
Supplement: Supplementary file 1 [file ijms-22-04004-s001.zip › Final supps/Supplementary Figure 3.pdf]

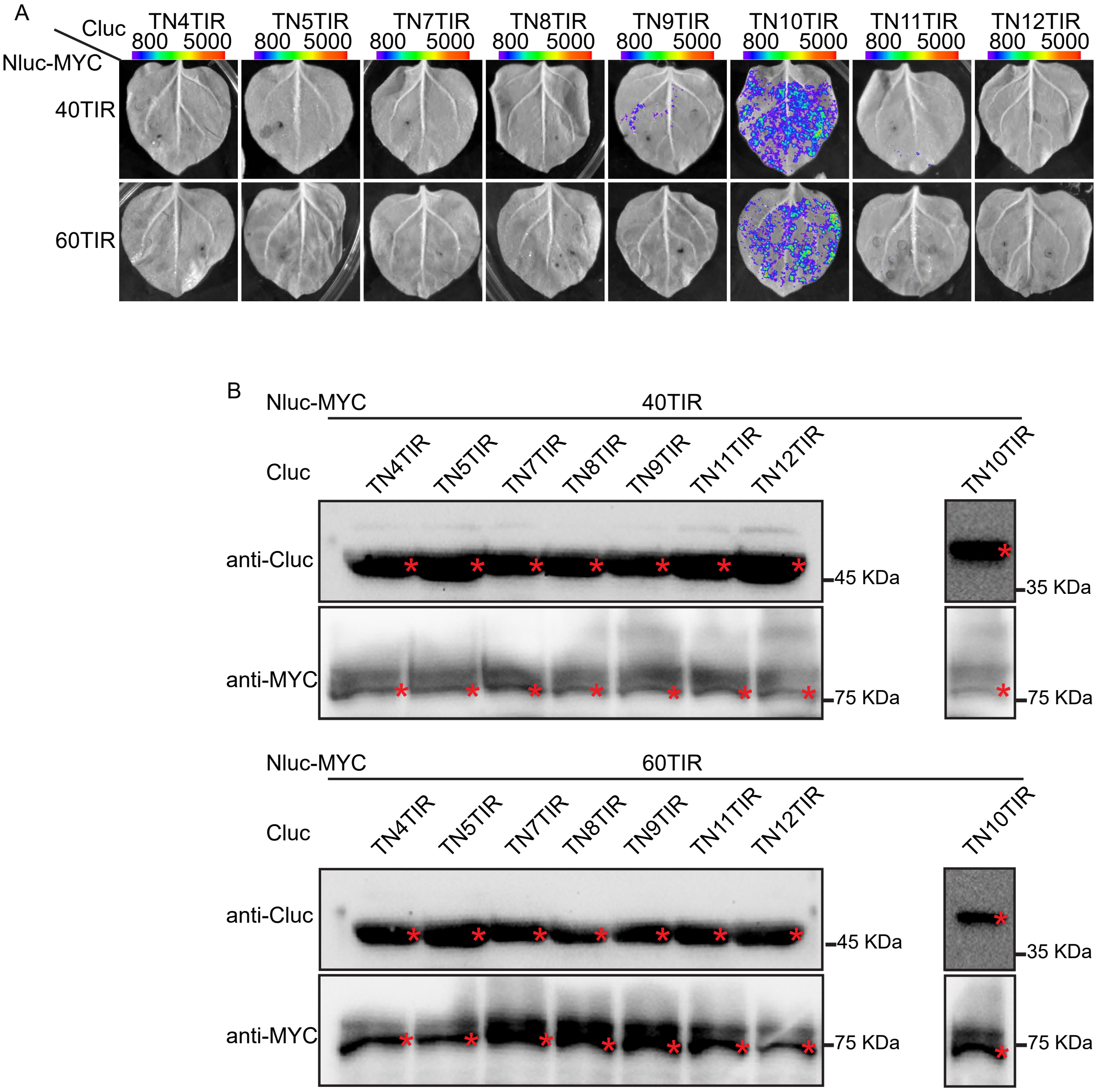


Supplementary Figure 3. Interaction detection between TNL40/60 TIR domain and TN4TIR-TN12TIR in LUC assays.
(A) The CDS of *TN4TIR*, *TN5TIR*, *TN7TIR*, *TN8TIR*, *TN9TIR*, *TN10TIR, TN11TIR* and *TN12TIR* were fused to the *Cluc*. *Agrobacterium* strain GV3101 carrying different construct was paired and injected into the *N. benthamiana* leaves. The infiltrated leaves were sprayed with 1 mM luciferin 48 hours after injection and the luminescence signal was captured by a cooled charge coupled device (CCD) camera.
(B) Immunoblotting analysis of co-expression 40TIR, 60TIR with TN4TIR, TN5TIR, TN7TIR, TN8TIR, TN9TIR, TN10TIR, TN11TIR, TN12TIR in *N.benthamiana*. the TNs-TIR proteins were detected by anti-Cluc and 40TIR, 60TIR were detected by anti-MYC. Asterisks (*) indicate the position of the corresponding proteins.
