# Supplementary material for: A Truncated TIR-NBS Protein TN10 Pairs with Two Clustered TIR-NBS-LRR Immune Receptors and Contributes to Plant Immunity in Arabidopsis
Source: Int J Mol Sci. 2021 Apr 13;22(8):4004. doi: 10.3390/ijms22084004 (PMC8069298; doi:10.3390/ijms22084004)
Supplement: Supplementary file 1 [file ijms-22-04004-s001.zip › Final supps/Supplementary Figure 4.pdf]

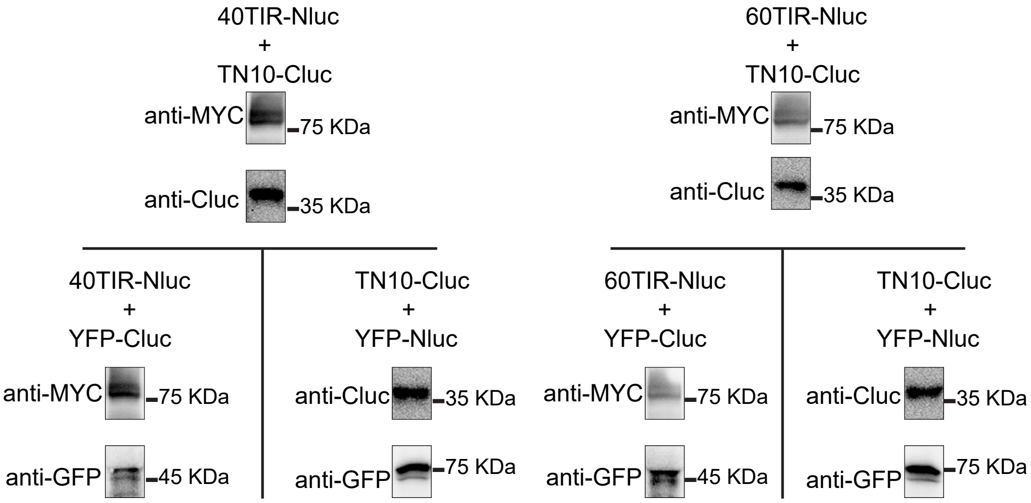

Supplementary Figure 4. Immunoblotting analysis of co-expression 40TIR-Nluc-MYC, 60TIR-Nluc-MYC with TN10-Cluc or YFP-Cluc, TN10-Cluc with YFP-Nluc in *N. benthamiana*.

Different pairs of constructs were transiently expressed in *N. benthamiana* leaves as described in Figure 2A. The TNL40TIR -Nluc-MYC, TNL60TIR-Nluc-MYC proteins were detected by anti-MYC, TN10-Cluc was detected by anti-Cluc, YFP-Nluc and YFP-Cluc were detected by anti-GFP
