# Supplementary material for: A Truncated TIR-NBS Protein TN10 Pairs with Two Clustered TIR-NBS-LRR Immune Receptors and Contributes to Plant Immunity in Arabidopsis
Source: Int J Mol Sci. 2021 Apr 13;22(8):4004. doi: 10.3390/ijms22084004 (PMC8069298; doi:10.3390/ijms22084004)
Supplement: Supplementary file 1 [file ijms-22-04004-s001.zip › Final supps/Supplementary Figure 5.pdf]

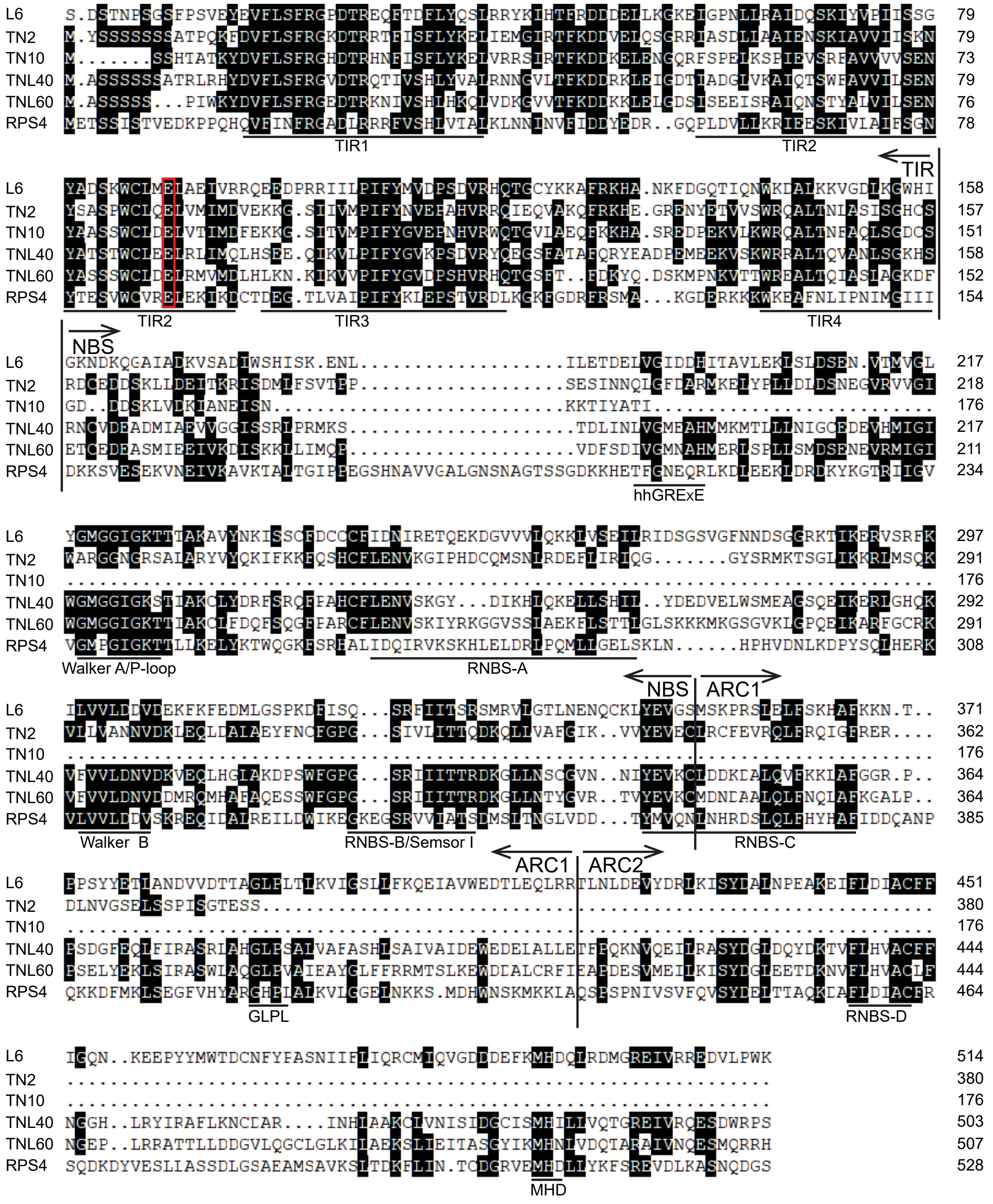

Supplementary Figure 5. TNL40, TNL60, and TN10 protein sequence alignments.
Amino acid sequences of L6 (positions 47-560), TN2, TN10, TNL40 (positions 1-503), TNL60 (positions 1-507) and RPS4 (positions 1-528) were aligned using DNAMAN software. Consensus amino acids (Homology ≥ 50%) are highlighted by black boxes. Structural characteristics are indicated above the sequence based on flax L6. The TIR, NBS, and ARC sub-domains are underlined. Boundaries between the TIR, NBS, and ARC domains are indicated by vertical black lines. The conserved glutamic acids (E) are labeled by a red box.
