# Supplementary material for: A Truncated TIR-NBS Protein TN10 Pairs with Two Clustered TIR-NBS-LRR Immune Receptors and Contributes to Plant Immunity in Arabidopsis
Source: Int J Mol Sci. 2021 Apr 13;22(8):4004. doi: 10.3390/ijms22084004 (PMC8069298; doi:10.3390/ijms22084004)
Supplement: Supplementary file 1 [file ijms-22-04004-s001.zip › Final supps/Supplementary Figure 6.pdf]

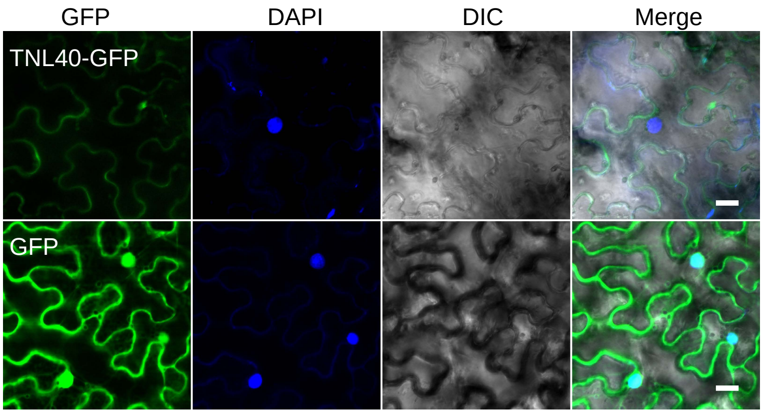

Supplementary Figure 6. Subcellular localization of TNL40 in *N. benthamiana* epidermal cells. The *TNL40-GFP* was transiently expressed in *N. benthamiana* leaves. GFP fluorescence was visualized 48 hours after transformation. The nucleus was displayed by diamidine phenylindole (DAPI) staining. The GFP alone protein was used as a control. Bar = 20 μm.
