# Supplementary material for: A Truncated TIR-NBS Protein TN10 Pairs with Two Clustered TIR-NBS-LRR Immune Receptors and Contributes to Plant Immunity in Arabidopsis
Source: Int J Mol Sci. 2021 Apr 13;22(8):4004. doi: 10.3390/ijms22084004 (PMC8069298; doi:10.3390/ijms22084004)
Supplement: Supplementary file 1 [file ijms-22-04004-s001.zip › Final supps/Supplementary Figure 7.pdf]

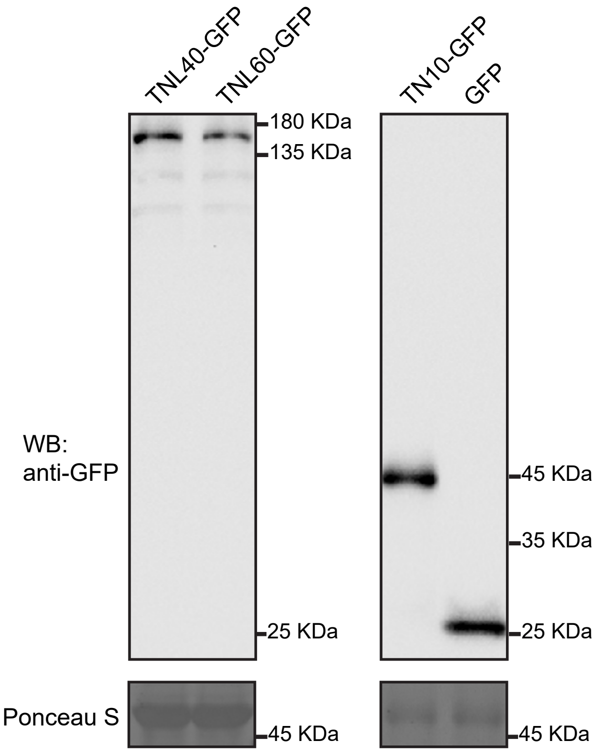

Supplementary Figure 7. Immunoblotting analysis of TNL40-GFP, TNL60-GFP, TN10-GFP and GFP in *N. benthamiana* leaves. *TNL40-GFP*, *TNL60-GFP*, *TN10-GFP* and *GFP* were transiently expressed in *N. benthamiana* leaves as described in Figure 4, the proteins were detected by anti-GFP antibody.
