# Supplementary material for: A Truncated TIR-NBS Protein TN10 Pairs with Two Clustered TIR-NBS-LRR Immune Receptors and Contributes to Plant Immunity in Arabidopsis
Source: Int J Mol Sci. 2021 Apr 13;22(8):4004. doi: 10.3390/ijms22084004 (PMC8069298; doi:10.3390/ijms22084004)
Supplement: Supplementary file 1 [file ijms-22-04004-s001.zip › Final supps/Supplementary Figure 8.pdf]

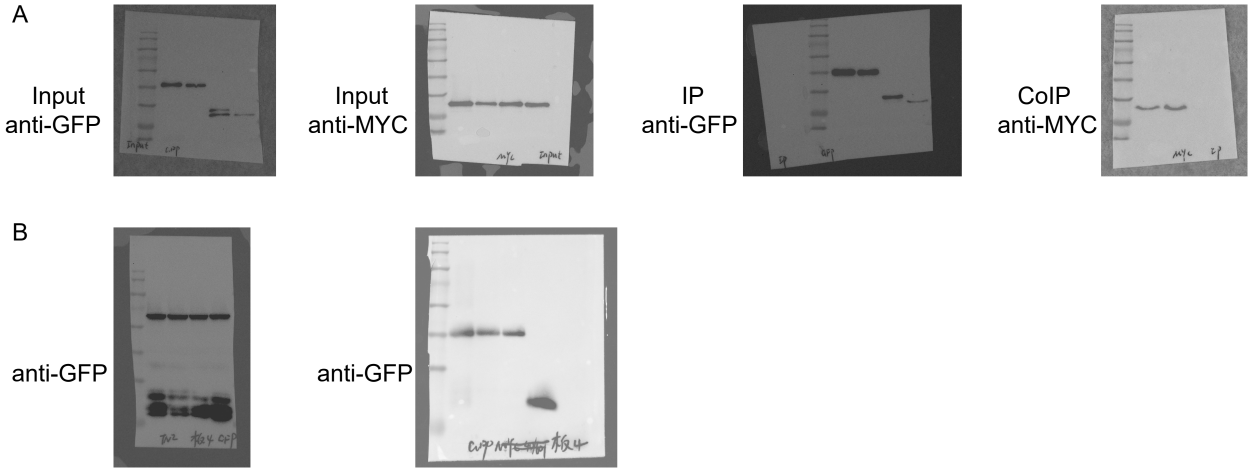

Supplementary Figure 8. The full size western blots gels of Figure 2B and Figure 5B
(A) The full size western blots gels of Figure 2B
(B) The full size western blots gels of Figure 5B
