# Supplementary figures and images for: A Truncated TIR-NBS Protein TN10 Pairs with Two Clustered TIR-NBS-LRR Immune Receptors and Contributes to Plant Immunity in Arabidopsis
Source: Int J Mol Sci. 2021 Apr 13;22(8):4004. doi: 10.3390/ijms22084004 (PMC8069298; doi:10.3390/ijms22084004)

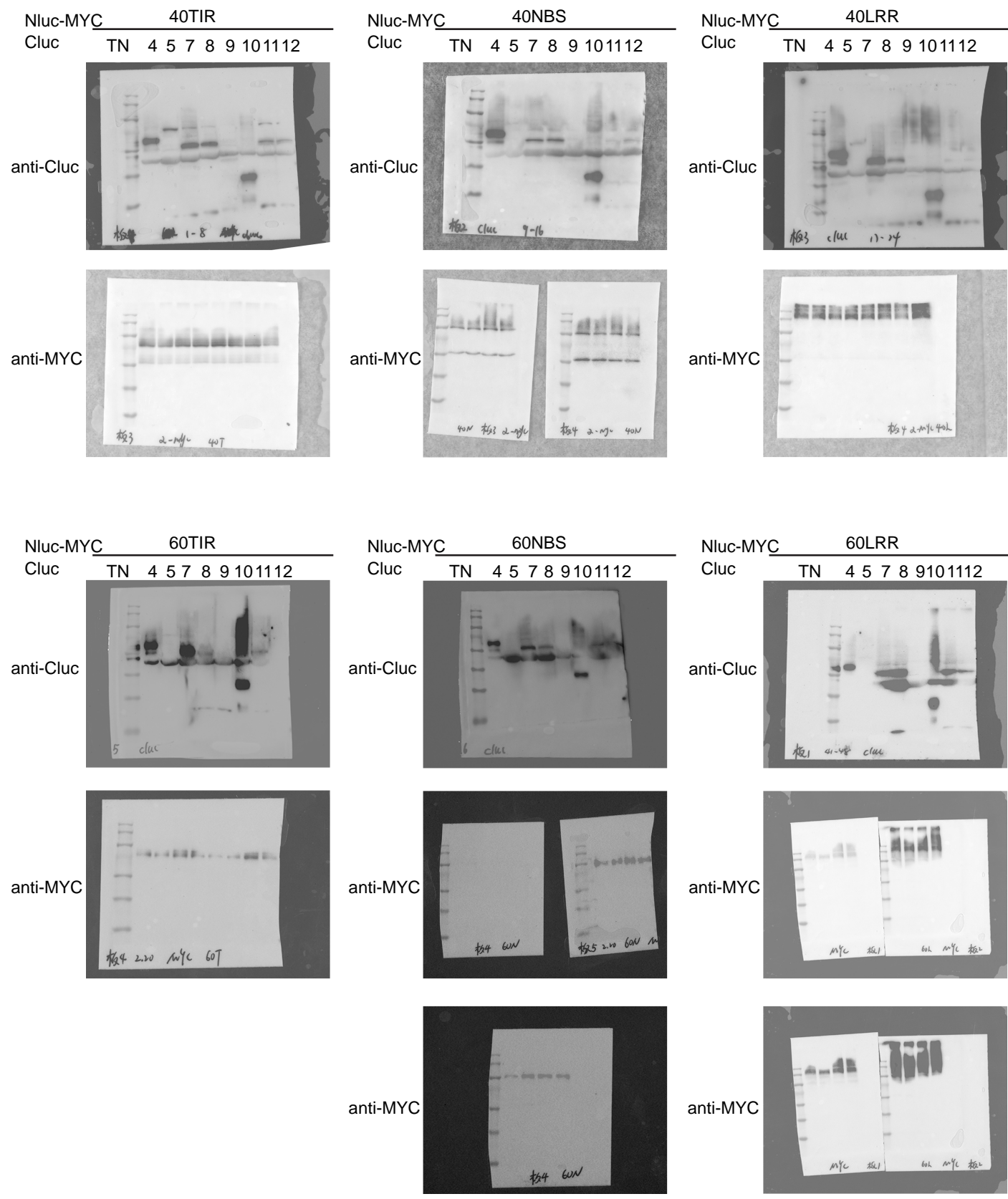

Supplementary Figure 9. The full size western blots gels of Supplementary Figure 2

Supplement: Supplementary file 1 [file ijms-22-04004-s001.zip › Final supps/Supplementary Figure 9.pdf]
